# Molecular changes in phenolic compounds in *Euglena gracilis* cells grown under metal stress

**DOI:** 10.3389/fpls.2023.1099375

**Published:** 2023-05-09

**Authors:** Eve Bernard, Céline Guéguen

**Affiliations:** Department of Chemistry, Université de Sherbrooke, Sherbrooke, QC, Canada

**Keywords:** cytoscape, metals, EC50,metabolites, polyphenol, HPLC-ESI-qTOF

## Abstract

Metal presence in the aquatic ecosystem has increased and diversified over the last decades due to anthropogenic sources. These contaminants cause abiotic stress on living organisms that lead to the production of oxidizing molecules. Phenolic compounds are part of the defense mechanisms countering metal toxicity. In this study, the production of phenolic compounds by *Euglena gracilis* under three different metal stressors (i.e. cadmium, copper, or cobalt) at sub-lethal concentration was assessed using an untargeted metabolomic approach by mass spectrometry combined with neuronal network analysis (i.e. *Cytoscape*). The metal stress had a greater impact on molecular diversity than on the number of phenolic compounds. The prevalence of sulfur- and nitrogen-rich phenolic compounds were found in Cd- and Cu-amended cultures. Together these results confirm the impact of metallic stress on phenolic compounds production, which could be utilized to assess the metal contamination in natural waters.

## 1 Introduction

The contamination of aquatic ecosystems has become a global concern (Feng et al., 2021) and can cause adverse impacts on aquatic ecosystems and human health (Stumm and Morgan, 1996). Some metals, such as Cu, and Co, play a significant role in the functioning of algae as an important component of enzymes and photosynthetic systems. These essential metals are necessary for growth and development, but elevated concentrations can have detrimental physiological effects on the physiology, morphology, and biochemistry of cells (Mathur et al., 2016; Deng et al., 2020; Nguyen et al., 2023). Their excess can cause adverse physiological effects. Other metals, such as Cd(II), are often reported in water bodies because of their numerous applications in industrial processes (Bulgariu and Bulgariu, 2005; Blanco-Vieites et al., 2022), but do not play any role in the cell metabolism process. Their presence can cause the inhibition of cell growth, disturbance of the photosynthesis process, and changes in cell permeability (Watanabe and Suzuki, 2002; Bergsten-Torralba et al., 2020; He et al., 2021).

One of the cellular responses to metal stress can induce the production of harmful oxidative molecules, such as free radicals and reactive oxygen species (ROS), which can upset the redox balance causing oxidative stress and leading to cellular damage and even apoptosis (León-Vaz et al., 2021). The defense system implements diverse mechanisms of action to regain homeostasis, which is crucial for normal cell activity. The production of exogenous and endogenous compounds with antioxidant properties, like glutathione, carotenoids, and polyphenols, can help scavenge oxidizing molecules (Poljsak, 2011; Pisoschi and Pop, 2015). The energy cost to synthesize them is relatively high but is necessary to preserve cell integrity and limit cellular damage.

Phenolic compounds often referred to as polyphenols, are secondary metabolites (Lindner and Pleissner, 2019) synthesized by organisms through the shikimate and phenylpropanoid metabolic pathways in response to external stimuli (Randhir et al., 2004; Sharma et al., 2019). These metabolites play an essential role in defense mechanisms against oxidative species (Naikoo et al., 2019). The presence of hydroxyl and carboxyl groups helps chelate metals and thus reduces oxidative stress (Michalak, 2006; Korzeniowska et al., 2020). There has been a growing interest in recent years for these important natural antioxidants and their chelating ability but only a few reports have focused on the analysis of bioactive phenolic compounds and their implication in algal growth under metal stress (Onofrejová et al., 2010; Rico et al., 2013).

Growth under metallic stress can increase the production of chelating metabolites (Alvilés et al., 2005; Holguin and Schaub, 2013; Mangal et al., 2019). Previous studies have observed an enhancement of the phenylpropanoid metabolic pathway, thus the number of phenolic compounds under various stress conditions (Michalak, 2006; Šamec et al., 2021). Cadmium (Cd) is a non-essential metal extremely toxic to living organisms causing significant negative effects on the diverse metabolic pathways, growth, and photosynthesis (Qian et al., 2009; Monteiro et al., 2011; Cerventes-Garcia et al., 2016) and is easily absorbed and accumulated by the organisms (Jamers et al., 2013; Griboff et al., 2018). Copper (Cu) is an essential metal used in numerous physiological processes but becomes toxic at higher concentrations (Gaetke and Chow, 2003; Rico et al., 2013) leading to DNA damage (Mittler, 2002; Knauert and Knauer, 2008). Cobalt (Co) is also an essential metal and a constituent of vitamin B12 required for cellular growth (Adam and Garnier-Laplace, 2003; dos Reis et al., 2021). In presence of high concentrations of Co(II), the syntheses of chlorophyll a and chlorophyll b (El-Sheekh et al., 2003; Fathi et al., 2008) and RNA (Nowicka, 2022) is disturbed.

This study aims to assess the molecular composition of phenolic compounds produced by the eukaryotic *Euglena gracilis* cells under metal stress (Cd, Co, Cu) using an untargeted metabolomic approach. The protist, *Euglena gracilis*, can tolerate elevated metal contaminations (ppm range; Zhang et al., 2016; Winters et al., 2017) *via* the production of a range of chelating metabolites that complex metals ions into a tolerable physiological form (Zenk, 1996; Khatiwada et al., 2020). An untargeted metabolomic approach will be used to assess the changes in the phenolic profiles in response to the metal stress.

## 2 Materials and methods

### 2.1 Cell growth and biomass harvesting

The *Euglena gracilis* strain CPCC469 purchased from the Canadian Phycological Culture Centre (University of Waterloo, Canada) was grown in pre-combusted 250 mL Erlenmeyer flasks containing 100 mL of modified acid medium (MAM; Bernard and Guéguen, 2022) supplemented with 5 g L^-1^ of glutamic acid at pH 3. The photoautotrophic cultivation was carried out at 25°C under illumination 16:8 h (light: dark) at 2800 Lumens Sylvania^®^ fluorescent lamp (2x F32W-GRO-LUX). The euglenoid cells in the exponential growth phase (initial cell density of 1.0 x 10^6^ cell mL^-1^; in biological duplicate) were exposed to a single metal solution (i.e., Cd(NO_3_)_2_,4H_2_O or Cu(SO_4_),5H_2_O or Co(NO_3_)_2_, 6H_2_O; SigmaAdrich) with nominal concentrations ranging from 4x10^-5^ to 20 ppm for Cd(II), from 3x10^-5^ to 130 ppm for Cu(II), and from 2x10^-5^ to 10^3^ ppm for Co(II). A triplicate control culture (no metal added) was also conducted under the same condition. A hemocytometer-based trypan blue assay and a light microscope (Leica DM500) were used to quantify living cells in each replicate. The metal concentration required to cause cell death in half of the cells (EC50) was calculated using OriginPro 2020.

To assess the molecular composition of phenolic compounds, *Euglena gracilis* cells were exposed to the EC50-72h of the metal. After 72h the biomass was harvested, centrifuged, thoroughly washed with Milli-Q water, and freeze-dried for subsequent analysis.

### 2.2 Phenolic compounds extraction and HPLC separation

The freeze-dried biomass (50 mg) was sonicated for 1h in an ultrasonic bath. The phenolic compounds were then extracted with a mixture of aqueous methanol (85%), 2,3-ter-butyl-4-hydroxyanisol (2 g L^-1^), and formic acid (0.1%). After centrifugation (4900 rpm, 30 min), the supernatant was evaporated with a rotary evaporator at 30°C. Resuspension was done in methanol (20%) and acidified water (1% formic acid) followed by filtration using a 0.2-µm polyethersulfone filter. Phenolic compounds were separated by gradient elution high-performance liquid chromatography (HPLC) coupled with a diode array detector. The chromatographic separation was performed according to Bernard and Guéguen (2022), using a reversed-phase column. Three chromatographic fractions were collected: 4.2-4.7 min (fraction 1; F1), 6.8-7.3 min (fraction 2; F2), and 10.8-11.3 min (fraction 3; F3) for MS analysis.

### 2.3 Mass spectrometry analysis

Catechin (*m/z* = 289.072; 4µmol L^-1^ in 50:50 v/v MeOH: Milli-Q water) was added to all the chromatographic fractions as an internal standard (**Figure S1**). The chromatographic fractions were then introduced into an electrospray ion source by direct infusion with a flow rate of 300 µL h^-1^ and ionized in a negative mode (source capillary voltage -5000 V). The MS analysis was done using a MAXIS time–of-flight mass spectrometer (Bruker). The signal acquisition was done across an *m/z* range of 100-1000 over 2 min lapse and in duplicate for each sample. An external standard (sodium formate) was used for instrumental calibration from day to day. MS grade methanol was infused between each sample to minimize sample carryover. A blank sample spectrum was also acquired every 10 samples and all *m/z* found in the blank were removed in the subsequent sample spectra. Spectral processing was conducted using Bruker compass DataAnalysis (v4.4) where a mass list was generated at S/N > 4 and a relative intensity cut-off of 0.1%. The formulas, corresponding to *m/z* within a 10 ppm mass error, were then attributed using the SmartFormula (DataAnalysis v4.4) with the following criteria: ^12^C(1-50), ^1^H(1-100), ^16^O(1-30), ^14^N(0-2), ^32^S(0-2). Only *m/z* present in both replicate samples was kept for the phenolic analysis. Phenolic compounds were defined as 0.6< H/C< 1.5 and 0.3< O/C< 0.85 (Kuhnert et al., 2013; Ngoc et al., 2019) and modified aromaticity index (AImod) inferior to 0.67 (Koch and Dittmar, 2016).

The abundance-based diversity (DA) values were calculated based on the Gini-Simpson index (Gotelli and Chao, 2013) and adapted to molecular mixtures (equation 1). It helps describe the distribution of phenolic compounds across formulas by considering the number of formulas identified and their relative intensity (Mentges et al., 2017). The index ranges from 0 to 1 and represents the probability that randomly chosen formulas to differ from one another (Mentges et al., 2017).


(Eq1) $$DA=1-\sum_{i=1}^{N}p_{i}^{2}\;$$

Where N is the total number of formulas in the data set, p_i_ represents the sum-normalized signal intensity of the i-th formula and i represents each of the formulas of the data set.

The different atomic ratios (O/C, H/C, S/C, and N/C) were calculated based on the formula attributed to each phenolic compound found in the different samples. The double-bond equivalent (DBE, equation 2) represents the sum of instaurations and rings in a molecule (Koch and Dittmar, 2016). The modified aromaticity index (AImod, equation 3) is used to calculate the potential heteroatom contribution, particularly oxygen, to the double bond in a molecule (Koch and Dittmar, 2016).


(Eq2) DBE=1+C−O−S− 1/2(N+H)

(Eq3) AImod= (1+C−1/2 O−S−1/2(N+H))/(C−1/2 O−N−S)

### 2.4 Metabolites network analysis

The network analysis was conducted using the Cytoscape software with the MettaNetter application (Longnecker and Kujawinski, 2016). This type of neuronal network analysis allows the visualization of HR-MS m/z and the quantification of their interactions (Jourdan et al., 2008; Smoot et al., 2011). Briefly, the complete list of phenolic *m/z* was imported into Cytoscape where each *m/z* was treated as a node (**Figure S2**). A pre-defined list of common metabolites found in living cells including chemical building blocks of organic molecules, such as amino acid and nucleic acid was also imported (**Table S1**; Longnecker and Kujawinski, 2016; Mangal et al., 2017). The exact mass differences between nodes were then calculated and defined as edges if they matched any of the listed metabolites within a ±2 ppm error (**Figure S2**). Networks of metabolites between *m/z* were then created and visualized. The relative abundance of each metabolite is reported here.

### 2.5 Statistical analysis

The Shapiro-Wilk normality test was performed followed by the Wilcoxon nonparametric (R studio). A significant difference was considered at the level of p< 0.05.

## 3 Results and discussion

### 3.1 Metal toxicity

**Figure 1:**
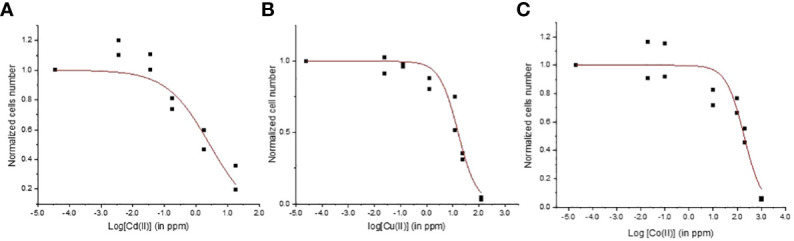
Concentration-response curves for Euglena gracilis cells exposed to **(A)** Cd, **(B)** Cu, and **(C)** Co.

Metal toxicity varies between different aquatic microorganisms species because of the variability of defense mechanisms and capacity to limit the metal concentration in cellular compartments thus restraining damages. The EC50 values for Cd, Cu, and Co were 2.7 ± 1.0 ppm, 16.0 ± 2.2 ppm, and 190.7 ± 39.9 ppm, respectively (r^2^ > 0.88; **Figure 1**). The toxicity of Cd for two microalgae *Chlorella elliposoidea* and *Raphidocelis subcapitata* microalgae were between 0.09 and 0.43 ppm (Magdaleno et al., 2014; dos Reis et al., 2021) whereas the EC50 of Cu^2+^ and Co^2+^ were reported to be 0.048 and 0.030 ppm for *Pseudokirchneriella subcapitata* (Al-Hasawi et al., 2020). The *E. gracilis* EC50 values were higher than the EC50 of other aquatic organisms confirming the good tolerance of *E. gracilis* to metal (Moreno-Sánchez et al., 2017). As expected, the lower EC50 values were found for the non-essential metal Cd whereas higher EC50 values were associated with the essential metal Co. Cd was reported to inhibit the synthesis of chlorophyll and carotenoids and the enzymes involved in CO_2_ fixation in many algal species (Nagajyoti et al., 2010; He et al., 2021; Mo et al., 2022) whereas the harmful effects for Cu and Co are related to the production of reactive oxygen species and lipids (Stauber and Florence, 1987; dos Reis et al., 2021).

### 3.2 Metal effects on phenolic compound production

**Figure 2:**
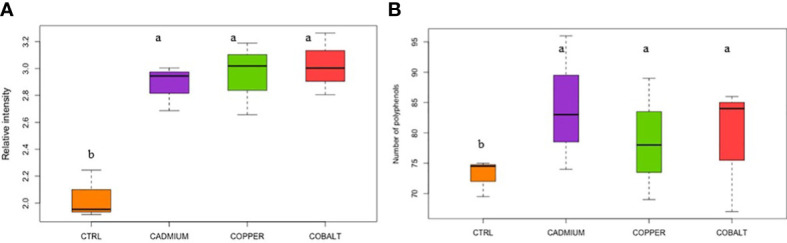
**(A)** Relative intensity and **(B)** number of cellular polyphenols in control (CTRL) and metal treatments. Different superscript letters indicate significant differences (p<0.05) determined by the Wilcoxon test.

The three chromatographic fractions (**Figure S4**) did not show any significant trends between fractions for the elemental ratios (O/C, H/C, N/C, and S/C) and indices (modified aromaticity index, double bond equivalent, nominal oxidation state of carbon). All phenolic compounds reported in this study are listed in **Supplemental Table S1**. Significant trends were found when the phenolic compound fractions (F1+F2+F3) were combined. The relative abundance and number of phenolic compounds were significantly lower in the control than in the metal cultures (p< 0.05; **Figure 2**). For example, the relative intensity of phenolic compounds accounted for 2.04 ± 0.11% in the control compared to 2.88 ± 0.10 and 3.02 ± 0.13 in metal cultures (**Figure 2A**). The metal exposure led to a significant increase in phenolic compound production for all metals, consistent with the implication of phenolic compounds in stress mitigation (Šamec et al., 2021). Interestingly, no significant differences were found in relative intensity and number of phenolics between the three metal treatments (p > 0.05). This means that exposure at EC50 values for metals leads to comparable concentration and abundance of phenolic compounds and thus similar cellular oxidative stress.

### 3.3 Molecular diversity of phenolic compounds

**Figure 3:**
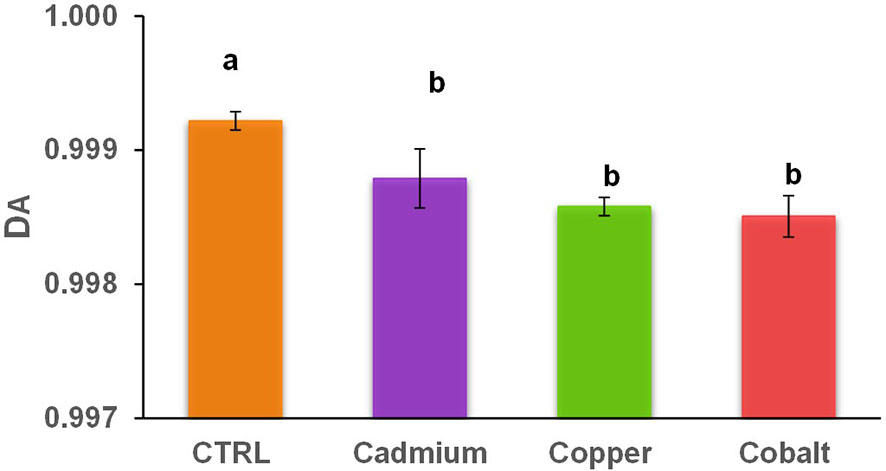
Abundance-based diversity (DA) of cellular phenolic compounds in control and metal treatments (cadmium, copper, and cobalt). Different superscript letters indicate significant differences (p<0.05) determined by the Wilcoxon test.

The abundance-based diversity (DA) of the metal cultures spanned from 0.9985 ± 0.0002 to 0.9988 ± 0.0002 which is significantly lower than in the control culture (0.9992 ± 0.0001; **Figure 3**). Larger values of DA indicate higher diversity, which means that the control culture had significantly higher diversity than the metal cultures. The phenolic compounds are involved in growth, cell division, and other activities but in a period of intense metal stress, cells are in survival mode pausing the majority of cell activities and favoring defense mechanisms (Michalak, 2006; Šamec et al., 2021). In contrast, the control culture showed a greater phenolic DA to maintain a variety of cellular processes for which less specific phenolic compounds are required. Since DA ranges from 0 to 1, it is possible to observe a great diversity of phenolic compounds produced by *E. gracilis* for all cultures (Caso and Gil, 1988; Rai and Kim, 2020). The diversity values were relatively high in all samples (over 0.99) compared to diversity values in previous population diversity studies of archaea, algae, and bacteria (0.1-0.96; Madigou et al., 2016; Liu et al., 2020).

**Figure 4:**
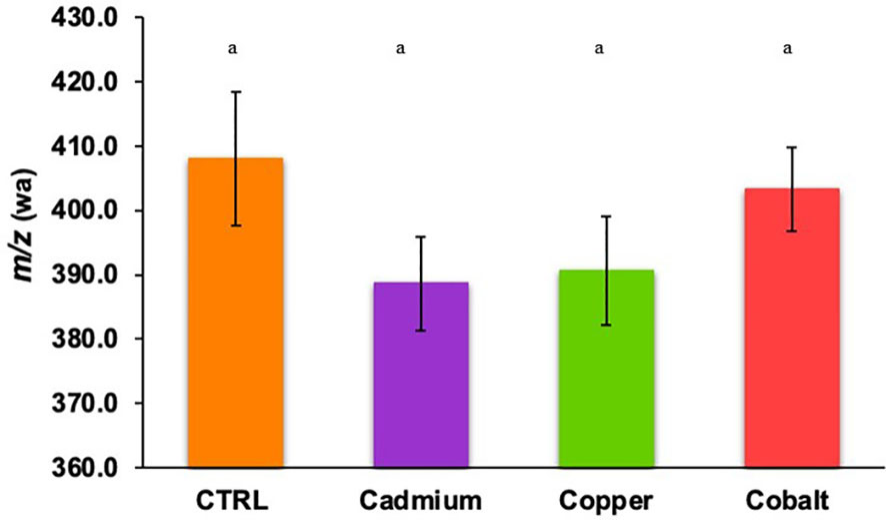
Weighted average *m/z* of cellular phenolic compounds found in control and metal treatments (cadmium, copper, and cobalt). Different superscript letters indicate significant differences (p<0.05) determined by the Wilcoxon test.

The weighted average *m/z* (*m/z*(wa)) of phenolic compounds was 408.04 ± 10.42 in the control culture and 388.67 ± 7.27 to 403.29 ± 6.47 in metal-stressed cultures (**Figure 4**). No significant difference in *m/z*(wa) was found between control and metal treatments, and between metal treatments, suggesting that the metal stress did not play a significant role in the *m/z*(wa) of produced phenolics. The stressed cells produced smaller phenolic compounds because the focus is to limit cellular damages caused by the exposition to metals causing oxidative stress. Thus, the energy is used for defense mechanisms instead of being used to produce complex metabolites (Hadacek and Bachmann, 2015; Anjitha et al., 2021; Feng et al., 2021).

### 3.4 Molecular characteristics of phenolic compounds under metallic stress

**Figure 5:**
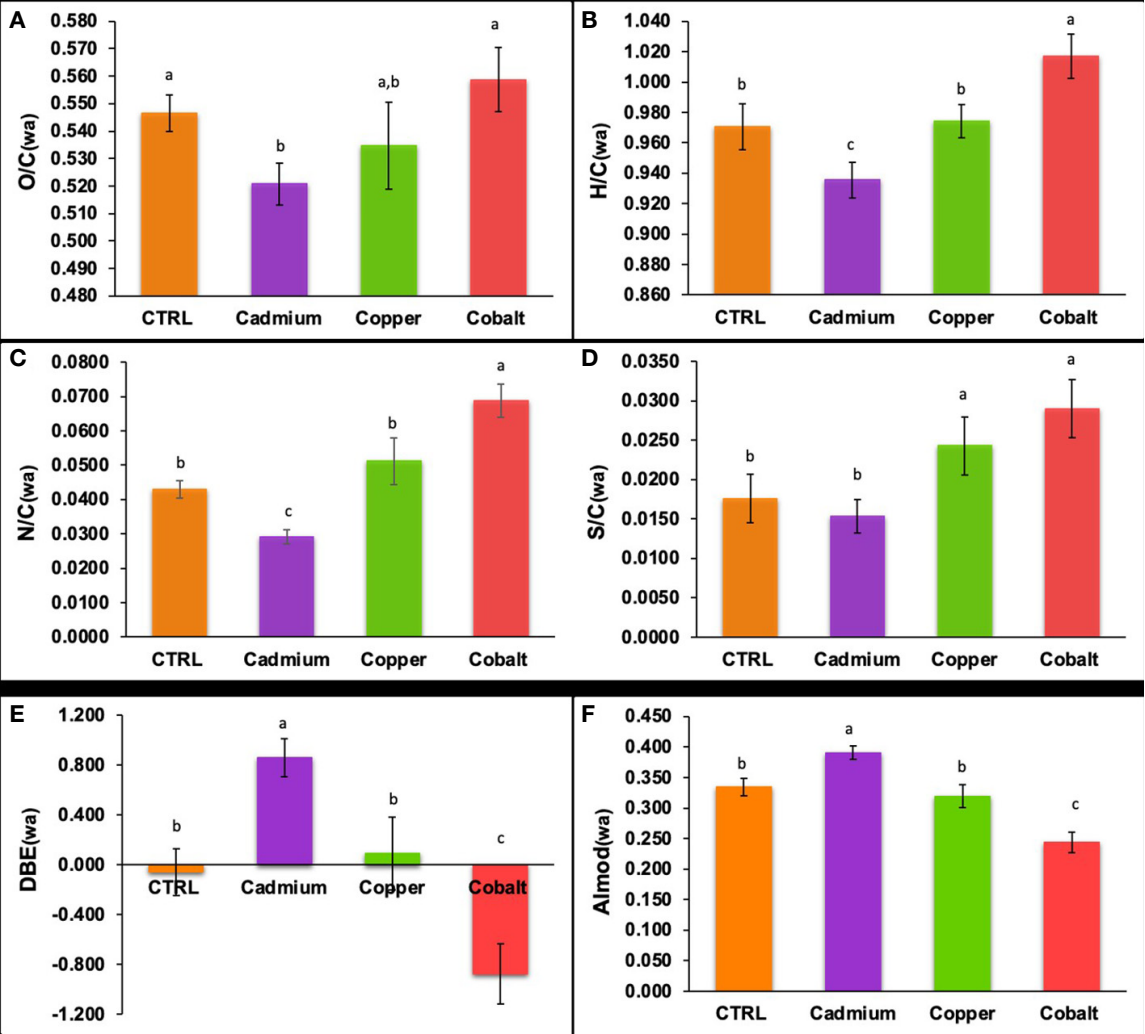
Weighted elemental **(A)** O/C, **(B)** H/C, **(C)** N/C, and **(D)** S/C ratios, **(E)** DBE, and **(F)** AImod of cellular phenolic compounds in control, cadmium, copper, and cobalt treatments. Different superscript letters indicate significant differences (p<0.05) determined by the Wilcoxon test.

In terms of elemental ratios (**Figures 5A-D**), significant differences in the elemental ratios of the phenolic compounds were found. The phenolic compounds had significantly lower O/C, H/C, and N/C ratios in the Cd-treatment than in the control (p< 0.05). The Co-treatment showed significantly higher H/C, N/C, and S/C ratios than the control (p< 0.05). The differences for the Cd-treatment suggested that the cellular phenolics were less oxygenated, more unsaturated, and N-poor in this treatment. The unsaturation was also confirmed by the higher DBE (0.86 ± 0.15, **Figure 5E**) and AImod (0.390 ± 0.011, **Figure 5F**) in the Cd-treatment. It is worth noticing that the cellular phenolic produced in the Co-amended cultures showed the opposite profile with a predominance of oxygenated (high O/C), saturated (high H/C), N enriched (high N/C) and S enriched (high S/C) compared to the control and Cu treatment. Together these results showed that the phenolic profile was metal specific.

### 3.5 Network analysis of phenolic compounds

**Figure 6:**
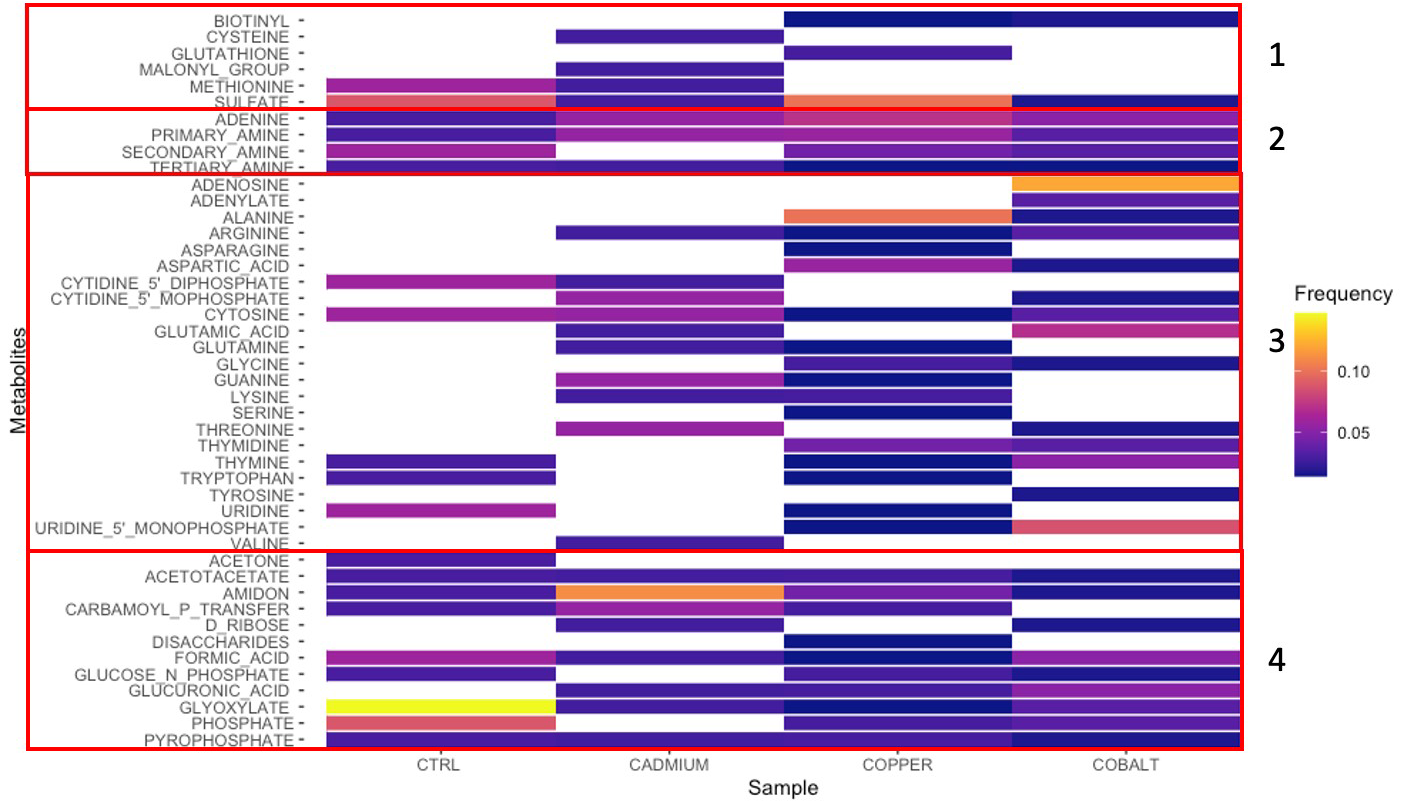
Metabolites found by network analysis corresponding to cellular phenolic compounds in control, cadmium, copper, and cobalt treatments. Groups 1-4 represent sulfur-, nitrogen-, nitrogen- and oxygen-, and oxygen-rich metabolites, respectively. The relative intensity is indicated by the color scale.

The *Cytoscape* network-based pathway analyses found 21 to 33 different metabolites in the control and stressed cells (**Figure 6**). The metabolites were grouped based on the presence of heteroatoms: sulfur (Group 1), nitrogen (Group 2), nitrogen and oxygen (Group 3), and oxygen (Group 4) (**Figure 6**). The metal-exposed cultures showed a greater abundance of heteroatoms containing metabolites (Groups 1, 3, and 4) because of the specific affinity for a metal type (Sten Ahrland et al., 1958). The type B metal, Cd, is known to promote sulfur-containing ligands, like cysteine and methionine (Group 1; **Figure 6**). Cobalt, a type-A metal, favors oxygen-containing metabolites (Group 3 - 4; **Figure 6**). Copper presents a less distinct profile favoring metabolites from all groups (**Figure 6**), which may be explained by the fact that this metal presents a different classification depending on its oxidation state (Duffus, 2002).

Glyoxylate (Group 4), a metabolite, involved in the anabolic metabolic pathway derived from the TCA cycle occurring in plants, bacteria, protists, and fungi (Chew and Than, 2021), is found in both control and metal treatments. The greater abundance of glyoxylate in the control cells confirmed that the metabolic machinery was optimal. Its reduced abundance in metal-stressed cells suggests that the defense mechanisms pathway was put on hold on other basic activities. Another metabolite found in all four cultures is adenine (Group 2), one of the four chemical bases in DNA. Unlike glyoxylate, adenine was more frequently found in metal-exposed cultures. Adenine, is one of the 2 nitrogenous bases of purine, with its derivatives (ATP, NAD, FAD) involved in cellular respiration and protein synthesis (Glavin et al., 2018). Allantoin, a purine intermediary metabolite can enhance abiotic stress tolerance *via* the activation of abscisic acid metabolism (Watanabe et al., 2014). Abscisic acid is a phytohormone implicated in normal growth and development in addition to playing a crucial role in integrating stress signals and controlling downstream stress signals (Tutreja, 2007). This may explain why adenine, *via* its intermediary, occurred more frequently in stress cultures than in the control.

## 4 Conclusion

The study assessed the molecular characteristics of phenolics produced by the eukaryotic *E. gracilis* when exposed to toxic levels of metal. The cellular extracts of metal-stressed cultures presented a significantly higher phenolic abundance than in the control culture, likely the result of the activation of defense mechanisms and the pausing of normal cellular activities. The molecular diversity in phenolic compounds determined by the Gini-Simpson index was significantly reduced to favor syntheses of less complex metabolites involved in metal stress management. The total number of phenolic compounds was similar between the three metal treatments meaning that the inherent metal toxicity did not influence the number of phenolic compounds but rather their molecular composition. The metal stress resulted in significant changes in the elemental ratios, unsaturation levels, and heteroatom abundances of the cellular phenolic compounds. Understanding the impact of metal exposure on phenolic compounds production and composition help identify conditions favoring specific metabolites with specific molecular characteristics.

## Data availability statement

The original contributions presented in the study are included in the article/**Supplementary Material**. Further inquiries can be directed to the corresponding author.

## Author contributions

EB, research design, experimentation, drafting, and statistical analysis. CG, research design, supervision, and review. All authors contributed to the article and approved the submitted version.
