# Supplementary material for: Molecular changes in phenolic compounds in Euglena gracilis cells grown under metal stress
Source: Front Plant Sci. 2023 May 9;14:1099375. doi: 10.3389/fpls.2023.1099375 (PMC10203486; doi:10.3389/fpls.2023.1099375)
Supplement: Supplementary file 1 [file DataSheet_1.docx]

Supplementary Material

# Supplementary Tables

**Table S1**: Metabolites for the network analysis.

| Metabolites | Molecular formula | *m/z* |
| --- | --- | --- |
| Alanine | C3H5NO | 71.037114 |
| Arginine | C6H12N4O | 156.101111 |
| Asparagine | C4H6N2O2 | 114.042928 |
| Aspartic Acid | C4H5NO3 | 115.026943 |
| Cysteine | C3H5NOS | 103.009186 |
| Cystine | C6H10N2O3S2 | 222.013286 |
| Glutamic Acid | C5H7NO3 | 129.042593 |
| Glutamine | C5H8N2O2 | 128.058578 |
| Glycine | C2H3NO | 57.021464 |
| Histidine | C6H7N3O | 137.058912 |
| Isoleucine | C6H11NO | 113.084064 |
| Leucine | C6H11NO | 113.084064 |
| Lysine | C6H12N2O | 128.094963 |
| Methionine | C5H9NOS | 131.040485 |
| Phenylalanine | C9H9NO | 147.068413 |
| Proline | C5H7NO | 97.052764 |
| Serine | C3H5NO2 | 87.032028 |
| Threonine | C4H7NO2 | 101.047678 |
| Tryptophan | C11H10N2O | 186.07931 |
| Tyrosine | C9H9NO2 | 163.063329 |
| Valine | C5H9NO | 99.068414 |
| Acetotacetate (-H2O) | C4H4O2 | 84.02113 |
| Acetone (-H) | C3H5O | 57.034040 |
| Adenylate (-H2O) | C10H12N5O6P | 329.052522 |
| Biotinyl (-H2O) | C10H14N2O2S | 226.077600 |
| Carbamoyl P transfer (-H2PO4) | CH2ON | 44.013639 |
| Co-enzyme A (-H2O) | C21H33N7O15P3S | 748.096826 |
| Glutathione (-H2O) | C10H15N3O5S | 289.073246 |
| Malonyl group (-H2O) | C3H2O3 | 86.000394 |
| Adenine (-H) | C5H4N5 | 134.046671 |
| Adenosine (-H2O) | C10H11N5O3 | 249.086190 |
| Adenosine 5'-diphosphate (-H2O) | C10H13N5O9P2 | 409.01885 |
| Adenosine 5'monophosphate (-H2O) | C10H12N5O6P | 329.052522 |
| Cytidine 5' diphosphate (-H2O) | C9H13N3O10P2 | 385.007621 |
| Cytidine 5' monophsophate (-H2O) | C9H12N3O7P | 305.041288 |
| Cytosine (-H) | C4H4N3O | 110.035437 |
| Guanosine 5- diphosphate (-H2O) | C10H13N5O10P2 | 425.013769 |
| Guanosine 5- monophosphate (-H2O) | C10H12N5O7P | 345.047436 |
| Guanine (-H) | C5H4N5O | 150.041585 |
| Guanosine (-H2O) | C10H11N5O4 | 265.081104 |
| Deoxythymidine 5' diphosphate (-H2O) | C10H14N2O10P2 | 384.012372 |
| Thymidine (-H2O) | C10H12N2O4 | 224.079707 |
| Thymine (-H) | C5H5N2O2 | 125.035102 |
| Thymidine 5' monophosphate (-H2O) | C10H13N2O7P | 304.046039 |
| Uridine 5' diphosphate (-H2O) | C9H12N2O11P2 | 385.991636 |
| Uridine 5' monophosphate (-H2O) | C9H11N2O8P | 306.025304 |
| Uracil (-H) | C4H3N2O2 | 111.019452 |
| Uridine (-H2O) | C9H10N2O5 | 226.05897 |
| Formic Acid (-H2O) | CO | 27.99491 |
| Glyoxylate (-H2O) | C2O2 | 55.98983 |
| Inorganic Phosphate | P | 30.9738 |
| Phosphate | HPO3 | 79.966332 |
| Primary amine | NH2 | 16.018724 |
| Pyrophosphate | PP | 61.9475 |
| Secondary amine | NH | 15.010899 |
| Sulfate (-H2O) | SO3 | 79.95682 |
| Tertiary amine | N | 14.00307 |
| D-Ribose (-H2O) (ribosylation) | C5H8O4 | 132.042259 |
| Disaccharide (-H2O) | C12H20O11 | 340.10056 |
| Glucose-N-Phosphate (-H2O) | C6H11O8P | 242.019156 |
| Glucuronic Acid (-H2O) | C6H8O6 | 176.032088 |
| Monosaccharide (-H2O) | C6H10O5 | 162.05282 |
| Trisaccharide (-H2O) | C18H30O15 | 486.15847 |

## Supplementary Figures


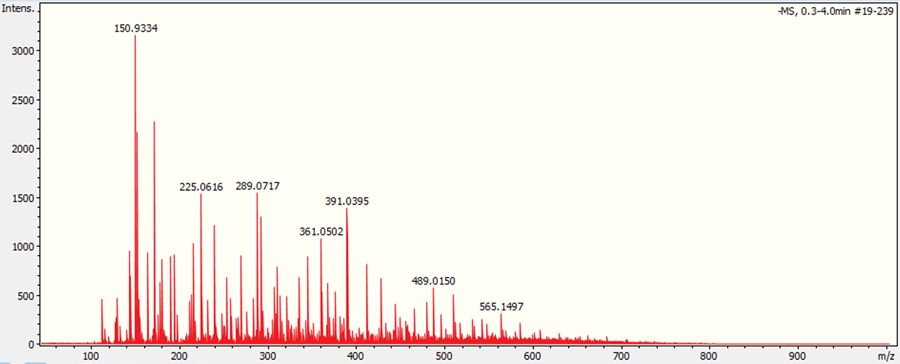


**Supplementary Figure 1.** MS spectra of a metal exposed culture with the catechin spike.


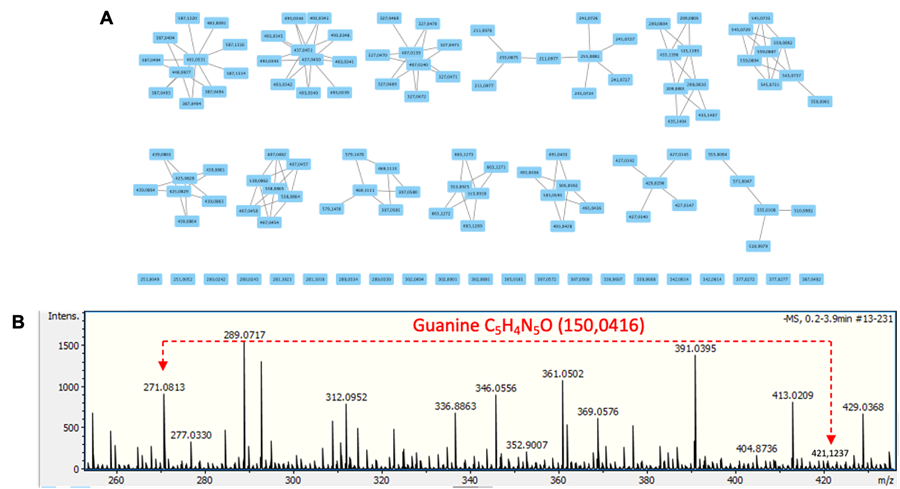


**Supplementary Figure 2**: **(A)** Nodes network and **(B)** metabolite identified by ∆ *m/z* from the network analysis.


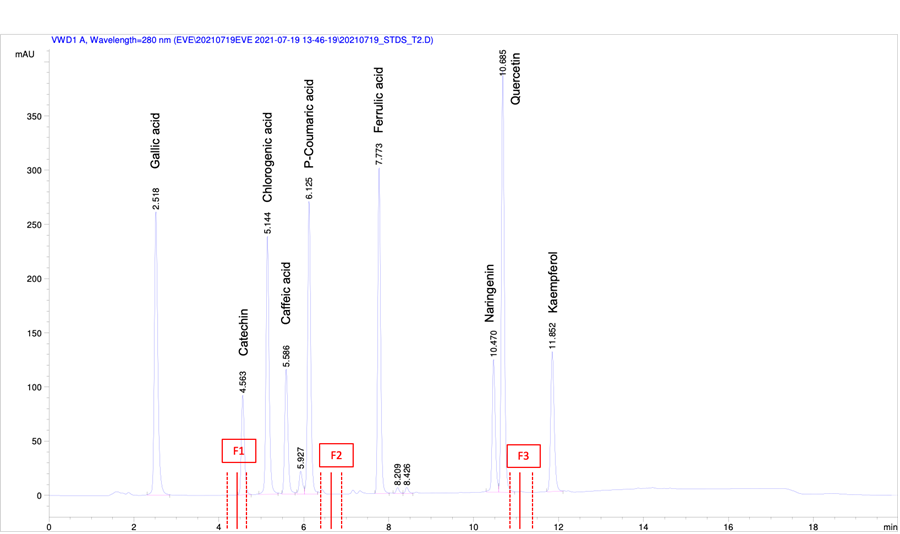


**Supplementary Figure 3**: Chromatogram of the phenolic standards. F1-F3 indicate the collected fractions in MS analyses.


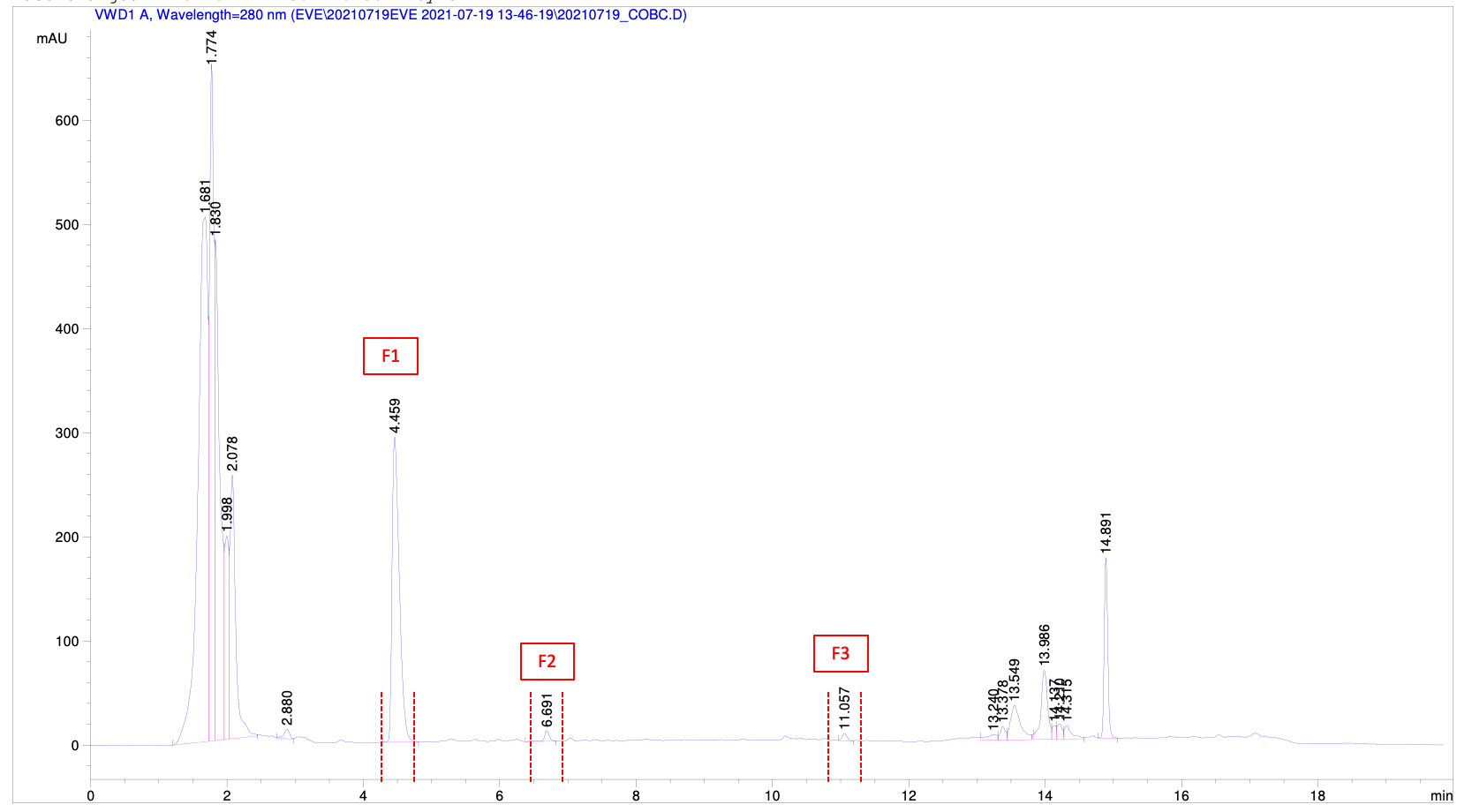


**Supplementary Figure 4**: Chromatogram of the 3 fractions in a cellular extract of a metal exposed culture. F1-F3 indicate the collected fractions in MS analyses.
